# Pediatric Stevens–Johnson syndrome associated with severe acute respiratory syndrome coronavirus 2 infection

**DOI:** 10.1111/ped.15366

**Published:** 2022-12-23

**Authors:** Shotaro Haraguchi, Shin Tsubokura, Koji Ozasa, Hiroyuki Sakuma, Shuichi Ito

**Affiliations:** ^1^ Department of Pediatrics Yokohama Rosai Hospital Yokohama Japan; ^2^ Department of Pediatrics Yokohama City University Graduate School of Medicine Yokohama Japan

**Keywords:** child, Kawasaki disease, multisystem inflammatory syndrome in children, SARS‐CoV‐2, Stevens–Johnson syndrome

Stevens–Johnson Syndrome (SJS), a life‐threatening disease characterized by high fever and severe mucocutaneous lesions, is triggered by drugs or infection. Multisystem inflammatory syndrome in children (MIS‐C) and Kawasaki disease can develop after severe acute respiratory syndrome coronavirus 2 (SARS‐CoV‐2) infection.^1^ There have been several cases of SJS reported in adults after SARS‐CoV‐2 infection,^2^ but few cases have been reported in children.^3^, ^4^ We experienced a pediatric case of SJS during SARS‐CoV‐2 infection and report that differentiating SJS from MIS‐C or Kawasaki disease was important.

A previously healthy 14‐year‐old boy developed flu‐like symptoms 7 days prior to admission, and he was diagnosed with SARS‐CoV‐2 infection. He was initially treated with cefcapene pivoxil, tipepidine hibenzate, carbocisteine, tranexamic acid, acetaminophen, and fexofenadine. His flu‐like symptoms including fever had resolved 4 days prior to admission. Nevertheless, he developed recurrent fever, ocular conjunctival hyperemia, and had a sore throat 2 days prior to admission to our hospital.

Upon admission, his vital signs were as follows: body temperature 38.1 °C, heart rate 117/min, blood pressure 110/53 mmHg, respiratory rate 20/min, and SpO_2_ 99% in room air. He presented with ocular conjunctival hyperemia, labial swelling, and erosions of the oral mucosa. Blood tests showed no abnormal findings except for slightly elevated inflammatory markers (white blood cell count 11 300/μl, C‐reactive protein 19.1 mg/L). Real‐time polymerase chain reaction (PCR) and an antigen quantification test with a nasopharyngeal swab for SARS‐CoV‐2 were both positive.

Initially, MIS‐C or Kawasaki disease were considered because of his mucocutaneous symptoms. A coronary artery echocardiogram was normal. The boy’s fever persisted after hospitalization and his mucosal lesions gradually exacerbated. On the fourth day of hospitalization, erythema multiforme with blisters were widespread on his trunk and upper and lower extremities (Figure 1). He suffered pain during urination because of urethral erosion. Taken together, we finally suspected he had SJS. An urgent skin biopsy revealed epidermal keratinocyte necrosis and intra‐ and sub‐epidermal blistering leading to a definitive diagnosis of SJS (Figure 1e).

**FIGURE 1:**
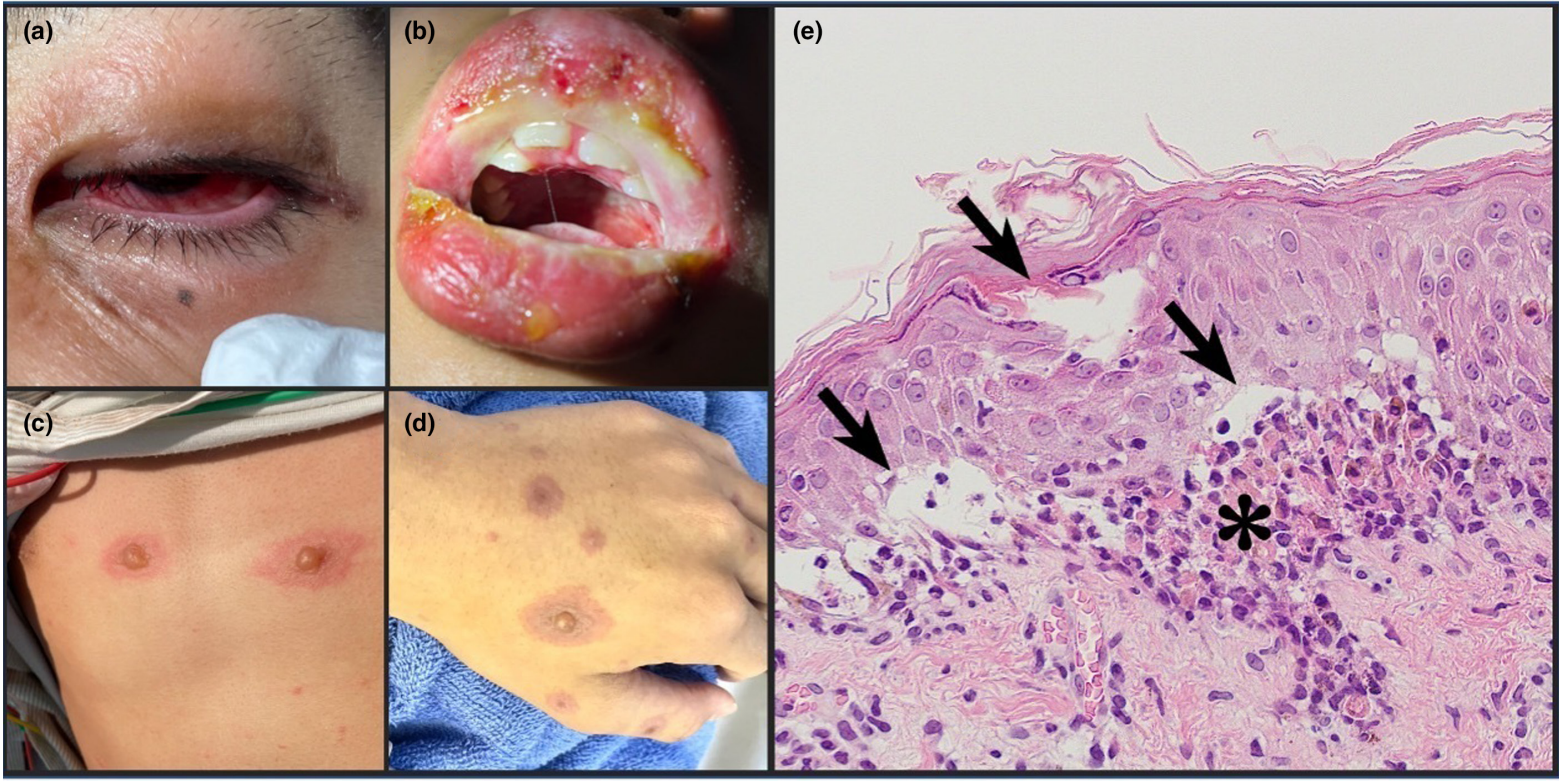
Skin and pathology findings. (a) Ocular conjunctival hyperemia. (b) Swelling and bleeding of the lips and oral mucosal erosion. (c), (d) Scattered exudative erythema multiforme on the chest, back, and upper and lower extremities with blistering in the center of the erythema. (e) Histopathological image of an area adjacent to skin eruptions on the upper arm (hematoxylin and eosin staining, ×200). Epidermal keratinocyte necrosis (asterisk) and sub‐epidermal blister formation (arrows) are shown.

Intravenous methylprednisolone pulse therapy (IVMP) followed by oral prednisolone were administered. The progression of skin and mucosal lesions was stopped by IVMP and these lesions gradually disappeared. No ocular lesions or respiratory involvement were observed as complications. Oral prednisolone was steadily tapered and the child was discharged without sequelae.

To determine whether SJS was triggered by infections other than SARS‐CoV‐2, we performed panel PCR analysis for multiple viruses in his blood after the diagnosis of SJS. Herpes simplex virus‐1 and ‐2, human herpes virus‐6, −7, and − 8, hepatitis B virus, Epstein–Barr virus, varicella‐zoster virus, Coxsackie virus, adenovirus, JC virus, cytomegalovirus, and human parvovirus B19 were all negative. A rapid test for group A streptococci and loop‐mediated isothermal amplification for *Mycoplasma pneumoniae* were negative. A drug‐induced lymphocyte stimulation test (DLST) 4 weeks after the onset of SJS was negative for all the drugs he was administered.

Stevens–Johnson Syndrome and toxic epidermal necrolysis (TEN) are often life threating because of secondary infection or organ involvement, especially the respiratory tract, gastrointestinal tract, and liver. Early diagnosis and intervention are therefore essential. As SARS‐CoV‐2 triggers MIS‐C and Kawasaki disease, we initially suspected these diseases. However, an urgent skin biopsy helped the definite diagnosis of SJS in our case. Epidermal keratinocyte necrosis and intra‐ and sub‐epidermal blister formation are specific pathological findings of SJS/TEN. A skin biopsy also helped differentiate SJS from MIS‐C or Kawasaki disease. The early diagnosis of SJS in our patient therefore allowed the early and successful treatment of disease with corticosteroids.

Stevens–Johnson Syndrome and TEN can be triggered by drugs and infections. Various infectious agents, including *Mycoplasma pneumoniae*, are associated with SJS in children,^5^ but there have been few reports of SJS associated with COVID‐19 – only two detailed cases of SJS/TEN complicated by COVID‐19 in children have been reported to date.^2^, ^3^ In previous reports, patients were treated with corticosteroids, immunoglobulin, and cyclosporine. In all cases, treatment was successful and no sequelae were observed. Our patient received multiple medications prior to the onset of symptoms. However, DLST for the drugs administered were all negative. These results suggest that his SJS may have been triggered by SARS‐CoV‐2 infection rather than drugs, although the sensitivity and specificity of DLST is not sufficiently high to definitely prove this. Further case studies should be accumulated to elucidate the pathogenetic mechanism of SJS.

## AUTHOR CONTRIBUTIONS

Shotaro Haraguchi, Shin Tsubokura treated the patient. Shotaro Haraguchi wrote the article with support from Shin Tsubokura and Koji Ozasa, and Shuichi Ito, and Hiroyuki Sakuma critically reviewed the manuscript. All authors read and approved the final manuscript.

## CONFLICT OF INTEREST

The authors declare no conflict of interest.

## INFORMED CONSENT

Informed consent was obtained from the patient and his parents for publication of this case report and accompanying images.
